# The Formation of Crystal Defects in a Fe-Mn-Si Alloy Under Cyclic Martensitic Transformations

**DOI:** 10.1186/s11671-016-1349-1

**Published:** 2016-03-09

**Authors:** Vladimir I. Bondar, Vitaliy E. Danilchenko, Viktor E. Iakovlev

**Affiliations:** G.V. Kurdyumov Institute for Metal Physics, NAS of Ukraine, Vernadsky Blvd. 36, Kyiv, 03142 Ukraine

**Keywords:** Martensitic transformation, Phase hardening, Dislocation, Stacking fault, Nanofragments, Diffraction

## Abstract

Formation of crystalline defects due to cyclic martensitic transformations (CMT) in the iron-manganese Fe-18 wt.% Mn-2 wt.% Si alloy was investigated using X-ray diffractometry. Conditions for accumulation of fragment sub-boundaries with low-angle misorientations and chaotic stacking faults in crystal lattice of austenite and ε-martensite were analyzed.

## Background

Cyclic martensitic transformations in iron-based alloys lead to formation of specific structural and phase state with a complex system of crystalline defects referred to as phase hardening [1]. Electron microscopy and X-ray diffractometry have shown that γ-α-γ (fcc-bcc-fcc) transformations in iron-nickel alloys result in an increase in dislocation density in reverted austenite more than by three orders of magnitude. Owing to high degree of phase hardening (after tens of repetitive transformations), γ-phase structure incorporates a large number of deformation twins. In case of multiple cyclic martensitic transformations (CMT), additional sub-boundaries are formed in these alloys, provided that generation of new dislocations, their accumulation, and interaction occur during cycling. This leads to the development of highly dispersed misoriented fragments of reverted austenite and triggers the process of phase recrystallization of the initial structure (nanofragmentation) [2, 3]. In contrast to iron-nickel alloys, the dislocation density of the reverted austenite in iron-manganese alloys, where γ-ε-γ (fcc-hcp-fcc) martensitic transformations occur, increases by no more than an order of magnitude [1]. This behavior can be explained when taking into account rather different volume effects of martensitic transformations. The γ-α and the γ-ε transformations are accompanied by the increase in the specific volume of 3 to 4 and of 1.75 %, respectively [4]. This difference causes the development of different numbers of dislocations in the process of the transformations. Again, iron-manganese alloys possess low energy of stacking faults that facilitates accumulation of chaotic stacking faults (CSF) during multiple CMT. Therefore, in alloys with different types of martensitic transformations, CMT result in formation of different types of crystalline defects.

## Methods

The intent of this work was to study the mechanism of accumulation of the misorientations and CSF in crystal lattice of austenitic and martensitic phases caused by cyclic γ­ε­γ martensitic transformations.

Our investigations were carried out in Fe-18 wt.% Mn-2 wt.% Si alloy with high completeness of γ-ε transformation (more than 90 % of martensitic ε-phase). This has enabled us to reach a high-scale phase hardening by multiple γ-ε-γ transformations.

The direct, γ-ε transformation in the alloy, occurred as the result of cooling in liquid nitrogen, and the reverse, ε-γ one, during consequent heating in a salt bath at the temperature of 380 °C. The cooling and the heating rates during the transformations were made as high as possible and reached 20°/s and 80°/s, respectively. Such mode of thermocycling inhibited relaxation processes and provided the effective accumulation of structural defects as a result of the direct and the reverse transformations. Such defects are capable to influence significantly on diffusion processes in phase hardened alloys.

The X-ray measurements were conducted on single crystal specimens exposed to Fe K_α_-radiation in RCW-86 rotation chamber. A modified URS-55 diffractometer with photoregistration was used. Single crystalline samples were selected as they enable us to observe misorientations of the crystal lattice in the range from a fraction of a degree to several tens of degrees. Also, they enable to observe the development of the structural fragmentation and refinement. The maximum misorientation angle, *ψ*, which characterizes grain fragmentation, was determined from azimuthal smearing of the (200)_γ_ reflections of austenite and the (002)_*α*_ reflections of martensite in diffraction pattern of single-crystal specimens.

## Results and discussion

###  Mechanisms of Misorientations Accumulation in Crystal Lattice of Austenite and ε­Martensite

**Fig. 1:**
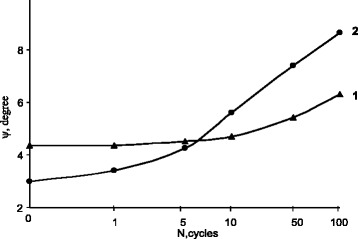
Maximum misorientation angle *Ψ* of the ε-martensite (*1*) and the austenite (*2*) versus number of γ-ε-γ cycles

In contrast with iron-nickel alloys, no signs of formation of such structural defects as high-angle boundaries of austenitic grains were observed in iron-manganese alloys [5, 6]. But the multiple γ-ε-γ transitions in the alloy studied resulted in the development of low-angle dislocation sub-boundaries. They can be characterized by *ψ*, the maximum misorientation angle of the crystal lattice. *ψ* grows monotonically with increasing the number of the γ-ε-γ transformations, up to 6.4° and 8.7° for the ε- and the γ-phases, respectively (Fig. 1).

After the first five γ-ε-γ cycles, the magnitude of *ψ* for the martensite was found to be higher than that for the austenite. Throughout this initial cycling, no visible accumulation of misorientations was observed, due to high crystallographic reversibility of the forward γ-ε and the reverse ε-γ transformations. But after 10 cycles of the transitions, misorientations started to accumulate in the crystal lattice of the austenite as well as in the martensite. When number of cycles is below 70–80, we are able to compare the magnitudes of *ψ* for the γ- and ε-phases. But after it exceeds 100, only the ε-phase can be registered using X-ray diffractometry. This is because, in the phase-hardened alloy, the γ-phase fully transforms into the ε-phase on cooling to room temperature after the completion of the reverse ε-γ transformation. The modification of the diffractometer used in this work was designed for room temperature measurements only; hence, after 100 cycles, the determination of *ψ* for the γ-phase was beyond the scope of this instrument. The accumulation of misorientations was continuously in progress up to 500 repeated transformation cycles.

The abovementioned mechanism of the accumulation of misorientations in crystal lattice of the ε-martensite points to a significant difference between degrees of the phase hardening attained due to the repeated γ-α-γ transformations, on the one hand, and due to the repeated γ-ε-γ transformations, on the other. In [1], it was concluded that phase hardening of metastable iron-nickel alloys can increase only if each of the subsequent γ-α-γ transformation cycles involves additional volume fraction of austenite. In this experiment, the degree of phase hardening of the iron-manganese alloy continued to grow after 100 γ-ε-γ transformations, though with every subsequent cycle volume fraction of austenite phase showed no change. This was evident from an appreciable increase in the *ψ* magnitude for the ε-phase as the number of the γ-ε-γ cycles was increased to 500. Meanwhile, the diffraction pattern of the iron-manganese alloy in this state was free of the γ-phase reflexes at room temperature.

The misorientation of the austenite lattice after the initial 5–7 cycles exceeded that of the ε-martensite. The higher values of *ψ* for austenite point to a suppression of the γ-ε martensitic transition in the most misoriented fragments of the reverted austenite. It has been known that the barrier effect of cellular (fragment) sub-boundaries on the growth of martensitic crystal is always weaker than that of grain boundaries and can be determined from the size of fragment and the misorientation angle between neighboring fragments [7].

An earlier electron microscopy revealed that α-martensite crystals overcame several sub-boundaries of fragments with low-angle misorientation but were stopped by sub-boundaries of supercritical misorientation [7]. The latter had an influence on growth of martensite crystal similar to the grain boundaries. The barrier effect of misoriented sub-boundaries in the α-martensite was higher than that in the ε-martensite. Indeed, after 3–5 γ-α-γ cycles, the difference between misorientation angles for crystal lattices of the γ- and the α-phases was 3°, whereas it did not exceed 6° for crystal lattices of the γ- and the ε-phases after 100 corresponding cycles. This difference characterizes distinction of dislocation structure of sub-boundaries, formed by the cyclic γ-α-γ and the γ-ε-γ martensitic transitions. In its turn, this enables to determine different degrees of influence of the sub-grains on diffusion characteristics in phase-hardened alloys.

As a result of the multiple γ-ε-γ cycles, the magnitude of *ψ* turned out to be considerably lower than in iron-nickel alloys after repeated γ-α-γ cycles. This is caused by higher structural reversibility of the forward γ-ε and the reverse ε-γ transformations. It is worth noting that even after intensive γ-ε-γ cycling, *ψ* did not exceed 9°, what is below the lower limit for the misorientation angle of the high-angle grain boundaries (14–15°). This implies that only low-angle sub-boundaries of fragments can be formed by the cyclic γ-ε-γ transformations and, in contrast to the iron-nickel γ-α-γ transformations, the γ-ε-γ ones are not able to form new grains of reverted austenite with different orientations with respect to the initial grain due to the accumulation of misorientations.

### Mechanisms of CSF Accumulation in Martensitic and in Austenitic Phases

The measurements of CSF concentration were performed on polycrystalline samples using automated DRON-3 diffractometer. CSF concentration in fcc austenite lattice was determined from the mutual shift of the Bragg (200)_γ_ and (111)_γ_ reflections [8–10]. In hcp lattice of the ε-martensite, CSF concentration was obtained using broadening reflections which satisfy the conditions: h − k = 3N ± 1 and l ≠ 0 (N—integers) [9, 11, 12].

The cyclic γ-ε-γ transformations resulted in introduction of CSF on the {111}_γ_ and the {001}_ε_ crystallographic planes of the reverted austenite and the ε-martensite of iron-manganese alloys with low energy of SF. The CSF in fcc and hcp structures have shown a tendency to accumulation [4]. The mechanisms behind these processes in the austenite and the ε-martensite of the phase-hardened Fe-18 wt.% Mn-2 wt.% Si alloy are still unclear and need to be investigated. In order to calculate the broadening of the ε-martensite peaks, the (100)_ε_ reflection was chosen as a reference since its half-width does not depend on CSF. Certain changes in the Fe-18 wt.% Mn-2 wt.%Si alloy diffraction pattern indicated the CSF accumulation on the austenite (111)_γ_ and the ε-martensite (100)_ε_ planes.

**Fig. 2:**
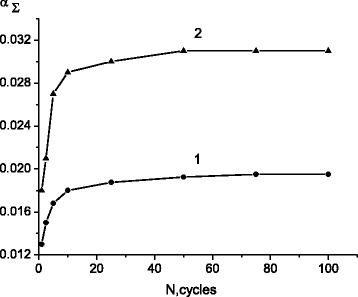
Variation of CSF concentration in the austenite (*1*) and in the ε-martensite (*2*)

The CSF concentration, α, in austenite grows rapidly with increasing the number *N* of the γ-ε-γ transformations to 10. After 50 cycles, α grows to 0.02 and further increasing *N* up to 100 only slightly increases α (Fig. 2, curve 1).

The CSF concentration in the ε-martensite also grows with *N* (Fig. 2, curve 2). The total CSF concentration (that is, taking the twin and the deformation SF together) reached a significant value of 0.032.

The formation of CSF in austenitic and martensitic phases suggests that fast cycling gives rise to introduction and accumulation of disordered SF, randomly distributed over fcc lattice. In certain regions of fcc structure with critical CSF concentration, there occurred an ordering of CSF and transformation of these regions from austenite to ε-martensite.

Electron microscopy revealed that the austenite matrix between plates of the ε-martensite was filled with stacking faults on [111]_γ_ planes, which facilitated the rearrangement of the fcc structure with accumulated CSF into the hcp structure and provided a specific mutual orientation between SF and the plates of the hcp phase. The authors of work [13] notice that concentration of SF in residual austenite increases with changing the temperature or applied stresses.

In the interval of the ε-γ reverse transformation, a drop of CSF concentration with decrease in amount of the ε-martensite was observed in austenitic and martensitic phases.

In order to gain some insight in to the change of the CSF concentration during annealing of the phase-hardened alloys, 200 γ-ε-γ cycles were performed. This resulted in accumulation of numerous CSF (with concentration of about 0.035) in ε-martensite. Heating the alloy to temperatures below the start martensitic temperature of ε-γ transformation did not lead to a decrease in the CSF concentration. But the CSF concentration decreased appreciably in the course of ε-γ transition, and finally, the stacking faults were eliminated when approaching the end of the transformation. Therefore, CSF as a by-product of phase hardening brought about by repetitive γ-ε-γ transformations can be removed in the process of annealing.

## Conclusions

The accumulation of misorientations in crystal lattice of the γ- and the ε-phases of the Fe-18 wt.% Mn-2 wt.% Si alloy due to the cyclic γ-ε-γ transformations proceeds less intensively than in iron-nickel alloys with γ-α-γ transformations. This difference follows from higher reversibility of the forward γ-ε and the reverse ε-γ transitions. Angle *Ψ* for the reverted austenite even upon intensive γ-ε-γ cycling did not exceed 9°–10°, which was lower than 14°–15° characteristic of high-angle boundaries. It can be concluded that in iron-manganese alloys, only low-angle sub-boundaries of initial grain fragments are formed during the cyclic γ-ε-γ transformations. The accumulation of misorientations in lattice of fragments could not lead to formation of new grains of the reverted austenite with orientations different from those of the initial grain, as opposed to iron-nickel alloys after γ-α-γ transformations.
